# Advances in Risk Factors for Recurrence of Common Bile Duct Stones

**DOI:** 10.7150/ijms.52974

**Published:** 2021-01-01

**Authors:** Yao Wu, Chen Jing Xu, Shun Fu Xu

**Affiliations:** 1 Sir Run Run Hospital, Nanjing Medical University, Nanjing, 211100, China; 2 Jiangsu Province Hospital, Nanjing Medical University, Nanjing, 210029, China

**Keywords:** common bile duct stones, ERCP, recurrence, risk factors

## Abstract

Choledocholithiasis is a chronic common disease. The incidence of cholelithiasis is 5%-15%, of which 5%-30% are combined with Choledocholithiasis. Although endoscopic cholangiopancreatography (ERCP) + endoscopic sphincterotomy (EST) is the most common treatment procedure, which clearance rate is up to 95%, the incidence of recurrent choledocholithiasis was 4%-25%. The risk factors of recurrence after choledocholithiasis clearance are the focuses of current researches, which are caused by multiple factors. We first systematically summarize the risk factors of common bile duct stones (CBDS) recurrence into five aspects: first-episode stone related factors, congenital factors, biological factors, behavioral intervention factors, and the numbers of stone recurrence.

## Introduction

**Figure 1:**
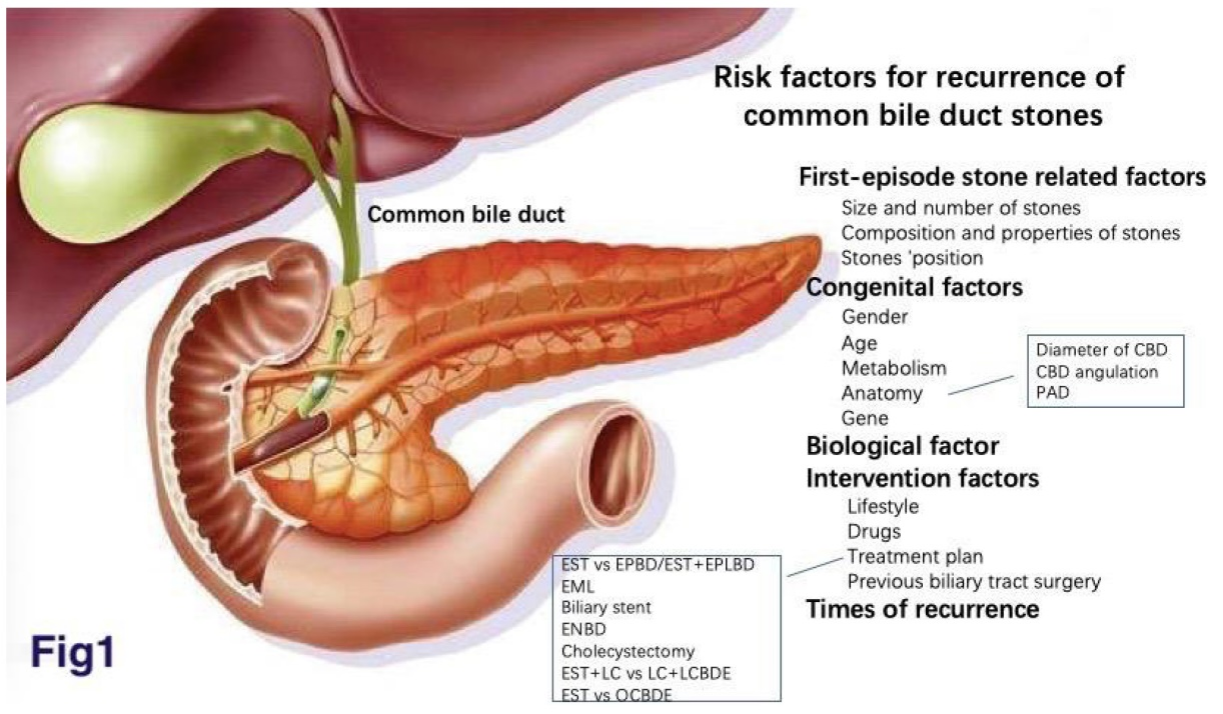
The recurrence factors of choledocholithiasis

Choledocholithiasis, one of the most common digestive diseases, is a chronic recurrent hepatobiliary disease whose pathological bases are impaired cholesterol, bilirubin, and bile acid metabolism. The incidence of cholelithiasis is 5% to 15%, in which the incidence of Choledocholithiasis is about 5%-30%.^19^, ^71^ Age, gender, genetic factors, factors related to metabolic syndrome, dietary factors ,and drugs are all risk factors for Choledocholithiasis.^43^ At present, endoscopic cholangiopancreatography (ERCP) + endoscopic sphincterotomy (EST) is the most common treatment procedure. ERCP intubation success rate is up to 98%, the clearance rate is up to 95%.^25^, ^55^ However, a large number of follow-up observations showed that the recurrence rate of common bile duct stone after endoscopic treatment was 4%-25%.^60^, ^65^ Choledocholithiasis may cause acute suppurative cholangitis, pancreatitis, biliary perforation, etc. The recurrent factors of choledocholithiasis are complex and cannot be explained by a single factor. Therefore, this article reviews the recurrence factors of choledocholithiasis (Fig. 1).

There is no uniform standard for the definition of the recurrence time. It is commonly considered that the recurrence of choledocholithiasis is defined as 6 months after the complete resection of primary stones.^39^, ^45^, ^77^

## Related factors of first-episode common bile duct stones

### Size and number of stones

Feng Deng et al. 19 found that the diameter of choledocholithiasis ≥10 mm was an independent risk factor for the recurrence of choledocholithiasis after ERCP. The larger the stone diameter, the larger the bile duct dilatation. When the normal bile duct motor function is affected, cholestasis and bile duct bacterial infection are easy to be caused, which is conducive to the formation of bile pigment stones. At the same time, when the stones are too large to be removed, most of them need in vitro ultrasonic lithotripsy, which is also a reason for the recurrence of large stones. Eun Soo Yoo 96 believed that multiple choledocholithiasis (≥2) was a related factor for the recurrence. However, Cheon suggested that the number and size of stones were the risks of residual bile duct stones after EST, rather than the risk of recurrence of bile duct stones.^29^ Different outcomes may be related to the number of stones defined, the size of the diameter, and the treatment regimen taken.

### Composition and properties of stones

The chemical composition of bile under the physiological state is balanced, and it is not easy to form stones. Sugiyama followed patients after EST for up to 10 years and found that recurrent stones were all brown pigment stones. Bacteria, cholestasis and papillary stricture may play an important role.^58^ Muddy stones are more likely to remain in the common bile duct. Small stone fragments missed by cholangiography may be the cause of stone aggregation and recurrence.^6^ However, YOO found that there was no significant correlation between muddy stones and common bile duct stones.^96^ There may be limited data on the consistency of stone, such as whether it is muddy or hard.

### Stones' position

The common bile duct is divided into four sections: the upper, posterior, pancreatic, and inner duodenal wall. The lower stones cause more bile retention, resulting in greater biliary wall pressure and greater degree of damage. Meanwhile, the closer the stone to the Oddi sphincter, the more damage it will have to its function. These may aggravate the recurrence of common bile duct stones. But there are no studies, and we are analyzing them.

## Congenital factors

### Gender

We all know that women are more likely to develop choledocholithiasis, which is associated with higher estrogen levels and less exercise. The decreased contractile force of the gallbladder muscles leads to delayed cholestasis and the precipitation of cholesterol crystals. However, most of the studies believed that there was no significant correlation between gender and the recurrence of common bile duct stones.^39^, ^80^

### Age

The recurrence rate of choledocholithiasis in patients over 65 years old is as high as 30%.^26^ Parra-membrives Pablo found that age was the only independent risk factor, with 86.4% occurring over 65 years of age.^66^ Common bile duct (CBD) expansion, CBD angulation, and periampullary diverticulum (PAD) were all associated with the recurrence of calculi in elderly patients.^36^

### Metabolism

Metabolic syndrome-related factors, such as abnormal lipid metabolism, hypercalcemia, hyperuricemia, obesity, lack of physical activity, insulin resistance, diabetes, and nonalcoholic fatty liver are risk factors for the occurrence of choledocholithiasis.^43^ Since these related factors do not disappear after stone removal, we believe that they are also risk factors for the recurrence of choledocholithiasis.

### Anatomy

#### Diameter of common bile duct

Long-term dilatation of the common bile duct results in decreased retraction of the smooth muscle fibers, impaired function, and difficulty in bile excretion, which is easy to result in cholestasis and bacterial infection, and promote the formation of stones. 87 Patients with common bile duct diameter of 15mm or larger were more likely to have a recurrence of symptoms and the risk of recurrence of stones was almost four times higher than those with a diameter of 10mm or less.^67^ Although it has been confirmed that the diameter of common bile duct is a risk factor for the recurrence, the specific diameter has not yet been determined. Deng 19 and Park 65 believed 10mm, but Song 77 and Yoo 96 believe that the diameter of common bile duct≥15mm is a potential predictor after endoscopic stoning. It is worth mentioning that Jeon Jin et al. 31 found that there is an inverse relationship between restoration of CBD diameter within two weeks and CBD stone recurrence.

#### Common bile duct angulation

The common bile duct usually leans to the right toward the duodenum during descent, which is called angulation. The bile flow speed is inversely proportional to the Angle of the bile duct. The prolonged biliary drainage time causes bile concentration, thus leading to the increase of cholesterol saturation, the decrease of bile duct systolic function, and a series of reactions such as unsaturated bile cannot be excluded, creating conditions for the recurrence of bile duct stones.^37^ There is no consensus on relationship between the specific angle and the recurrence. Keizmam et al. 35 and Ryu Seongyul et al. (73)reported ≤145°, but Rongchun et al. believed ≤135°.^97^ Chong et al. found 130° was a special angle.^16^ The difference in Angle may be related to preoperative/postoperative tablet reading, which requires further big data study. At the same time, the current data is based on the two-dimensional plane, which is not as accurate as the three-dimensional plane.

#### Periampullary duodenal diverticulum (PAD)

Most of the PAD is located within 2-3cm of the duodenal nipple. PAD can be divided into three types: Type I: the nipple is located within the diverticulum; Type II: The nipple is located at the inner edge of the diverticulum; Type III: The nipple is located outside the diverticulum. 77 More than 75.5% of PAD cases occurred in patients older than 50 years.^54^ PAD goes hand in hand with the formation of the stones 52, 74, but it is still unclear what is the mechanism of PAD causing the recurrence. The possible mechanism is as follows: 1 the inverse vertical overgrowth of bacteria and its spread to the biliary tract system; meanwhile, food was deposited in the diverticulum, forming a good medium for the bacteria. 2 The periampullary diverticulum may affect the normal anatomy of the nipple, causing compression of distal duct and expansion of the upper duct. 3 Patients with elevated biliary pressure or biliary spasm may cause the Impaired bile outflow.^76^ The size and type of PAD are associated with diameter of CBD and recurrence of choledocholithiasis. Choledocholithiasis recurrence rates of PAD I type were higher than PAD II and III type.^79^ The mean age and common bile duct diameter of patients with PAD< 15 mm were lower than those with PAD size≥15 mm.^38^

### Gene

A study of 43,141 Swedish twins with gallstone disease found that about 25 percent of the risk of gallstone disease was genetically determined.^33^ Mutations in some genes may be responsible for the formation of gallstones.^7^, ^8^, ^44^ ABCB4 (encoding the hepatobiliary flippase) gene mutation has been confirmed to be the main genetic risk factor for gallstone recurrence.^72^ The ABCB4 gene is involved in encoding multi-drug resistance protein 3 (MDR3) of the hepatcholine transporter, and MDR3 damage leads to reduced levels of phosphatidylcholine and promoting the formation of stony bile. The ABCG5/8 (encoding the hepatobiliary cholesterol transporter 5/8) allele is interrelated with the recurrence of choledocholithiasis, and its variant gene ABCG D19H is currently recognized as a genetic risk factor for the formation of gallstones, which can effectively predict the recurrence of choledocholithiasis.^89^

## Biological factors

Exogenous β-glucuronidase produced by anaerobes can hydrolyze bilirubin to unbound bilirubin (UCB) and glucuronic acid. Bacterial production of phospholipase A1 hydrolyzes lecithin (phosphatidylcholine) to produce free palmitic and stearic acids. The conjugated bile salts can be hydrolyzed by anaerobes' conjugated bile salt hydrolysates to form insoluble free bile acids. The above products can be combined with calcium ions in bile to form insoluble precipitation, which becomes the core framework of brown stones.^10^, ^56^, ^88^, ^90^ The bacteriological and morphological studies of 38 brown stone specimens showed that the positive rate of bacterial culture was 80.5%, and enterococcus was the most common microorganism isolated.^46^ The inflammatory pathological changes of the bile duct epithelium can persist for a long time after the removal of bile duct stones. Therefore, we believe that preoperative biliary tract infection in patients with first-episode stones may affect the relapsing of common bile duct stones due to impaired biliary tract function.

Microbiota cause disease through dysbiosis and shift in local distribution. Chen et al.^12^ analyzed the bacterial 16S rRNA sequence. LEfSe analysis was used to further determine that there was a significant difference in abundance between patients with recurrent choledocholithiasis and patients with new choledocholithiasis. Patients with recurrent choledocholithiasis were mainly Aeromonas, Enterococcus, Unclassified_Enterobacteriaceae, and Citrobacter. The authors are conducting this study from different levels, such as phylum, class, order, family, genus and species, and we speculate that the mechanism of microflora causing stone recurrence might be the change of dominant microflora, rather than the change of the entire biliary population. However, whether certain microflora changes in the recurrent group are associated with the recurrence of choledocholithiasis remains to be further studied.

In recent years, helicobacter pylori, HBV, HCV, Clonorchis sinensis, ascaris, and schistosomiasis have also been found to increase the risk of recurrence of choledocholithiasis.^47^, ^51^

## Intervention factors

### Lifestyle

Some studies have shown that a typical diet high in calories, cholesterol, fatty acids, or carbohydrates increases the risk of gallstones. 18, 21, 84 Physical activity reduces biliary cholesterol saturation by increasing HDL-C 23 and by affecting plasma TG and insulin release 82. But there is no study on the influence of systematic classified dietary factors or exercise on the recurrence of choledocholithiasis.

### Drugs

UDCA (Ursodeoxycholic acid) may effectively prevent the recurrence of choledocholithiasis by promoting the dissolution of cholesterol stones and reducing cholestasis.^78^, ^91^ UDCA has a protective effect on recurrence after choledocholithiasis resection.^13^, ^93^ However, some cases have been reported that UDCA promotes stone recurrence, but the mechanism remains unknown. The occurrence of biliary and duodenal anastomosis and recurrent biliary tract infection is associated with the formation of UDCA stones.^2^ PPI (Proton pump inhibitor) is found as a hazard factor for choledocholithiasis relapsing after EST. The possible mechanism is that PPI may increase the risk of choledocholithiasis recurrence by promoting the overgrowth of bacteria in the small intestine and changing the bacterial mixture in bile.^28^

### Treatment plan

Nowadays, the treatment of patients with choledocholithiasis is relatively mature, and minimally invasive endoscopic treatment is the mainstream strategy. Some of the less widely used strategies include laparoscopic common bile duct exploration (LCBDE), open common bile duct exploration (OCBDE), dissolution, extracorporeal shock wave lithotripsy (ESWL), percutaneous radiation, electrohydraulic lithotripsy (EHL), and laser lithotripsy.^4^, ^48^, ^57^, ^61^, ^62^, ^69^

#### EST vs EPBD /EST+EPLBD

EST was applied in the treatment of choledocholithiasis in 1974, initiating a new era in the treatment. Actually, a study from 11 centers showed that EST was a risk factor for recurrence.(95)The possible reasons for the recurrence of choledocholithiasis after EST include bile duct inflammation, papillary stricture, choledochal dilatation, diverticulum, duodenal contents returning to the bile duct, foreign body in the bile duct, etc. EPBD is an alternative therapy for choledocholithiasis.^81^, ^85^ EPBD can retain 70% ODDI sphincter function, and has fewer biliary complications, improve long-term prognosis, and lower stone recurrence rate.^22^, ^41^ However, EPBD is not widely used because of its association with a higher incidence of pancreatitis and the use of mechanical lithotripsy.^50^, ^83^ Compared with simple EST, small incision EST combined with EPBD showed close success rate of treatment of common bile duct stones, but the recurrence rate of postoperative stones was significantly reduced and the need for lithotripsy was greatly reduced. (53)The smallest duodenal papillary incision could prevent the recurrence of choledocholithiasis.^59^ However, some studies showed that the recurrence rate of common bile duct stones after EST combined with large balloon dilatation of the nipple under endoscope corresponds to that of the simple EST or EPLBD.^40^ ^14^ This difference may be related to the size of the nipple incision and bile duct dilation.^42^ Kim 40 believed that the diameter of common bile duct ≥22mm was a risk factor, which needed more research and verification.

#### Mechanical lithotripsy under endoscope (EML)

For the bile duct is difficult to clear the huge stone, to be taken out after lithotripsy. EML may lead to residual small stone fragments in the common bile duct, which cannot be detected by cholangiography. These micro-stones form the core of the stone again, which becomes a hidden risk for the recurrence of choledocholithiasis. Lithotripsy was an independent risk factor for the recurrence of choledocholithiasis. If the stones were not removed completely during lithotripsy, a stent was used to drain enough cholestasis to reduce the recurrence of stones.^49^

#### Biliary stent

Biliary stenting is considered as an alternative choice to EST in the elderly.^1^, ^5^, ^17^ Ueda et al. treated 66 patients with acute obstructive cholangitis due to CBDS by biliary stent implantation, but not EST. They found that short-term stenting without EST eliminated choledocholithiasis while the duodenal papillary function is preserved.^86^ What's more, Choi Jung-Hye found that stenting for A short period (one month when liver function normalized) after ERCP can reduce the risk of stone recurrence.^15^ However, Kaneko et al.^32^ found that long-term (≥301 days) placement of plastic stents and bile duct dilation during stent implantation were independent factors for SSC (stent-stone complex) formation. Short-term use of biliary stents can fully drain bile and relieve cholestasis, reducing small residual stones, which can reduce the risk of common bile duct stone recurrence. However, long-term implantation can induce bacterial proliferation and promote the occurrence of stones.

#### Endoscopic nasobiliary drainage (ENBD) and saline preventive irrigation

ENBD after ERCP can reduce the recurrence rate of stones. Nasobiliary drainage can increase positive biliary pressure to promote biliary drainage and reduce biliary reflux. The pre-existing cholestasis and biliary inflammation can be cleared to a certain extent^94^, thus reducing the incidence of choledocholithiasis. Meanwhile, for multiple small stones that are difficult to remove, nasobiliary drainage helps to reduce residual stones. Alternatively, prophylactic saline irrigation of the biliary tract after ERCP may also reduce the recurrence of common bile duct stones.^24^, ^30^

#### Cholecystectomy

Cholecystectomy after ERCP is still a controversial topic. The2017 UK guidelines for the treatment of choledocholithiasis stated that: All the cholecystolithiasis or patients with gallstones should undergo cholecystectomy unless there are specific cases where surgery is not permitted.^92^ But in practice, it's not that simple.^27^ Partial recurrence of choledocholithiasis may result from gallbladder metastasis. Therefore, prophylactic cholecystectomy after ERCP treatment of choledocholithiasis could reduce recurrence.^34^ However, the gallbladder can excrete bile and flush the biliary tract to prevent the stones formation. This function is also lost when the gallbladder is removed. Park et al.^65^ showed a prior history of cholecystectomy can increase the risk recurrence of choledocholithiasis after LCBDE and reported that prophylactic cholecystectomy was not required to prevent recurrence of choledocholithiasis in Asian populations without stones after ERCP stone removal. YOO et al.^96^ found that cholesterol stones were a risk factor for the recurrence of choledocholithiasis after cholecystectomy. Accordingly, the diagnosis of primary or secondary choledocholithiasis is very important.

It's suggested that cholecystectomy should be considered carefully in elderly patients, as they have low relative risks of recurrence of stones and may have high surgical complications. 64 However, CBD expansion resulting from cholecystectomy in youth is another acquired risk factor for CBDS.^9^ Besides, early (usually one week) cholecystectomy after EST in patients with choledocholithiasis combined with gallbladder stones can prevent recurrent biliary complications.^70^, ^75^

#### EST vs LCBDE

Platt et al. reported that LCBDE had a higher clearance rate of choledocholithiasis than ERCP (96.7% and 90%, respectively).^68^ Some retrospective comparative analyses and meta-analyses showed that the rate of recurrence was lower in the LCBDE group compared to the patients who had an EST.^3^, ^63^ LC+LCBDE preserve the complete sphincter function of Oddi, which may be associated with the reduction of the recurrence rate of common bile duct stones. A larger sample size should be studied to clarify this conclusion.

#### EST vs OCBDE

Some clinicians believe that the recurrence rate of choledocholithiasis is higher after EST than after choledochotomy OCT. This may be associated with the occurrence of pancreatitis. However, there is little data on whether there is a difference between the two methods in the treatment of CBDS. Zhou et al.^98^ concluded that compared with OCT, EST was superior to OCT in terms of duration of relief of biliary obstruction, duration of anesthesia, duration of surgery, and hospitalization days. However, there was no significant difference between OCT and EST in the recurrence and mortality of choledocholithiasis. This result needs to be further verified because most critical and elderly patients prefer to choose minimally invasive EST. Therefore, larger sample studies are needed.

### Previous biliary tract surgery

Previous biliary surgery (such as bile duct exploration and T tube drainage) can damage the bile duct wall and form scars, resulting in biliary stricture and poor bile excretion. At the same time, adhesion causes bile duct tilt, and bile angulation affects bile flow, which promotes the recurrence of stones. Multiple ERCP operations also aggravate Oddi sphincter injury and increase the chance of retrograde infection.^20^, ^49^

## Times of recurrence

Park et al. 64 conducted an average follow-up of 4.2 years among 46,181 south Korean patients with endoscopic choledocholithiasis, and found 5228 patients (11.3%) with first-time recurrence of choledocholithiasis. The recurrence rate was low for the first time, high for the second and third time, 23.4 % and 33.4 %, respectively. It can be seen that the recurrence rate is directly proportional to the number of recurrence. Deng et al. conducted a follow-up study on 477 patients with recurrent choledocholithiasis and found that the recurrence rate was as high as 19.5 %for the second recurrence and 44.07%for the third or more recurrence.^19^ The possible reason is that multiple surgical treatments aggravate the injury of Oddi sphincter and induce the necrosis of bile duct epithelial cells, forming scars and leading to bile duct stricture. Local adhesions, resulting in bile duct angulation and cholestasis, lead to stone recurrence. Surprisingly, when Chang JH et al. 11 followed up 481 cases, they found that choledocholithiasis increased in the recurrence period, 10.1± 5.2mm at the initial onset, 13.5± 7.3mm at the first recurrence, and 16.8± 7.8mm at the second recurrence. Therefore, regular follow-up examination is suggested for those who have had a recurrence, and it is recommended to follow up once every 1-2 years.
